# Decomposition of Phosphorus Pollution and Microorganism Analysis Using Novel CW-MFCs under Different Influence Factors

**DOI:** 10.3390/molecules28052124

**Published:** 2023-02-24

**Authors:** Chunpeng Leng, Yonggang Yuan, Zhiyu Zhang, Qiushi Shi, Fuping Li, Hao Wang

**Affiliations:** 1 College of Mining Engineering, North China University of Science and Technology, Tangshan 063210, China; 2 Key Laboratory of Bioelectrochemical Water Pollution Control Technology in Tangshan City, North China University of Science and Technology, Tangshan 063210, China

**Keywords:** constructed wetland, domestic sewage, electricity production, microbial fuel cell, total phosphorus

## Abstract

A constructed wetland (CW)-coupled microbial fuel cell (MFC) system was constructed to treat wastewater and generate electricity. The total phosphorus in the simulated domestic sewage was used as the treatment target, and the optimal phosphorus removal effect and electricity generation were determined by comparing the changes in substrates, hydraulic retention times, and microorganisms. The mechanism underlying phosphorus removal was also analyzed. By using magnesia and garnet as substrates, the best removal efficiencies of two CW-MFC systems reached 80.3% and 92.4%. Phosphorus removal by the garnet matrix mainly depends on a complex adsorption process, whereas the magnesia system relies on ion exchange reactions. The maximum output voltage and stabilization voltage of the garnet system were higher than those of the magnesia system. Microorganisms in the wetland sediments and electrode also changed considerably. It indicates that the mechanism of phosphorus removal by the substrate in the CW-MFC system is adsorption and chemical reaction between ions to generate precipitation. The population structure of proteobacteria and other microorganisms has an impact on both power generation and phosphorus removal. Combining the advantages of constructed wetlands and microbial fuel cells also improved phosphorus removal in coupled system. Therefore, when studying a CW-MFC system, the selection of electrode materials, matrix, and system structure should be taken into account to find a method that will improve the power generation capacity of the system and remove phosphorus.

## 1. Introduction

The rapid development of economic construction has caused serious damage to the water ecology, making wastewater treatment an important issue [1,2]. Industrial wastewater not only consumes a large amount of water but also has a low reuse rate. Agricultural irrigation water and sewage discharges contain large amounts of pesticides and fertilizers which seep from the soil to the groundwater, resulting in serious degradation of the aquatic environment [3]. Even the electrochemical treatment of wastewater has attracted extensive attention [4,5,6]; a constructed wetland (CW)-coupled microbial fuel cell (CW-MFC) system can generate electrical energy and treat sewage [7,8], which can not only improve the power generation performance of the MFC, but also improve the removal efficiency of organic matter. Indeed, CW-MFC is a new sewage treatment system with very promising development prospects [9]. However, there are still many operational problems to be solved.

At present, biological and chemical phosphorus removal is the most used phosphorus removal method in China. However, the biological method produces large amounts of sludge, with high environmental requirements and changes in water quality which may significantly impact the phosphorus removal effect. The chemical method faces problems such as low adsorption saturation, pH-dependent reaction effect, and high cost. Wetland construction has been widely used as an ecological engineering facility with low investment, high efficiency, and easy maintenance which can effectively achieve phosphorus removal. In recent years, the construction of wetland systems for phosphorus removal has received widespread attention. It was shown that cyanobacteria are able to transfer electrons through nanowires [10].

The CW-MFC system is a new type of sewage treatment system [11,12]. The CW-MFC coupling system can generate electricity while treating sewage. The power generation performance of MFC and the removal rate of organic matter are improved. It is a promising new sewage treatment system [13,14]. Its unique working principle means that it can treat wastewater and generate energy at the same time, which has broad development prospects [15,16].

Phosphorus is the key nutrient that causes water eutrophication [17]. In recent years, there has been a considerable increase in the research on phosphorus removal with CW systems [18,19]. In this study, the CW-MFC system was taken as the research object, and CW-MFC wetland substrate selection, operating conditions, and electricity generation performance were investigated. Firstly, matrices with good phosphorus removal effects were screened out, and the influence of their related properties on phosphorus removal and electricity generation performance was studied. The CW-MFC system can effectively remove phosphorus while improving electricity generation. The changes and functions of microorganisms in the process of phosphorus removal in the CW-MFC system are discussed, and the mechanisms driving phosphorus migration, transformation, and removal in the CW-MFC system are analyzed. The results provide theoretical and technical support for the popularization and application of CW-MFC systems to remove phosphorus.

## 2. Results and Discussion

### 2.1. Analysis of the System Phosphorus Removal Effect

#### 2.1.1. Total Phosphorus Removal Efficiency at Different Pollutant Concentrations

In this set of experiments, four different influent phosphorus concentrations were used: 1, 2, 3, and 5 mg/L. The total hydraulic retention time was 5 days, and sampling took place once every 24 h. After filtration, the total phosphorus concentration in the top discharge solution was determined, and the effects of the magnesium and garnet treatment under different influent pollutant concentrations were compared. The garnet device was referred to as device I and the magnesia device as device II.

**Figure 1:**
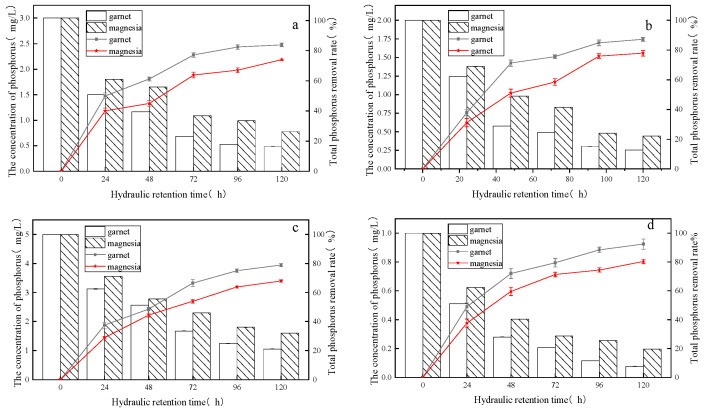
Total phosphorus concentrations and removal rates. (**a**) 1 mg/L; (**b**) 2 mg/L; (**c**) 3 mg/L; (**d**) 5 mg/L.

Figure 1 shows that when the phosphorus concentration of the influent water was 1, 2, 3, and 5 mg/L, the total phosphorus removal rate of device I reached 92.4%, 87.35%, 82%, and 78%, respectively, and the phosphorus removal rate for device II reached 80.3%, 77.9%, 75%, and 67%, respectively. The treatment effect of device I was stronger than that of device II. Furthermore, as the hydraulic retention time increases, the simulated domestic sewage treatment efficiency at the different influent phosphorus concentrations also shows an upward trend. When the hydraulic retention time was 1 day, the removal efficiency showed a rapid increase and the removal rates of the two devices reach about half of the total removal rate. The removal rate increased rapidly from the second day to the third day, but then the removal rate only increased slightly. This indicated that the removal of total phosphorus in the system mainly depends on filtration and adsorption of the substrate. When the substrate saturation reached a certain degree, the growth rate of the removal rate slowed. The total phosphorus removal rate also decreased as the influent pollutant concentration increased. The sewage treatment effect was optimal when the influent pollutant was 1 mg/L. In general, an increase in influent pollutant concentration and long-term operation of the system could easily saturate the matrix. However, the phosphate ions were spilled in this system followed by adsorption and degradation.

#### 2.1.2. Comparison between the Phosphorus Removal Effects of the CW-MFC and CW Systems

After the completion of the second phase of the experiment, the microbial fuel cell was disconnected and the external resistor, the cathode, and the anode were removed, which made the system into a separate CW system that still used the operation mode of water inlet and outlet from the bottom. The phosphorus concentrations in the water were 1, 2, 3, and 5 mg/L, respectively. The total hydraulic retention time was 5 days, and the systems were taken every 24 h. After filtration, the total phosphorus concentration in the topmost aqueous solution was determined, and the phosphorus removal effect was compared with that of the CW-MFC system.

**Figure 2:**
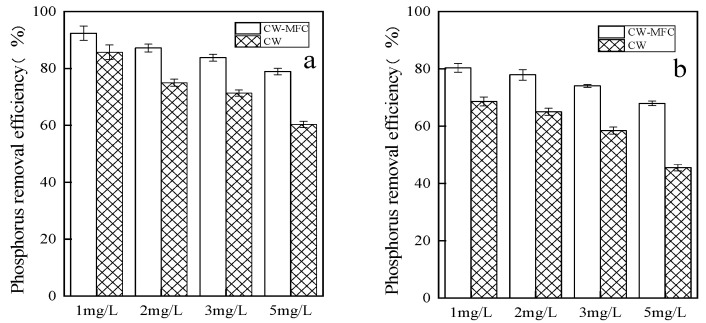
Phosphorus removal by the CW and CW-MFC systems. (**a**) Constructed system based on garnet; (**b**) constructed system based on magnesia.

Figure 2 was a comparison of the phosphorus removal effect between the CW-MFC system based on the two substrates and the CW system. It was found that, for both substrates, phosphorus removal by the CW-MFC system was better than that of the CW system. The difference between the two systems was smallest when the influent concentration was 1 mg/L. Furthermore, removal by the garnet-based system is better than that of the magnesia-based system. This shows that the type of MFC system has an impact on the removal of total phosphorus. In addition, the electrolysis of the system and the action of microorganisms such as phosphorus-accumulating bacteria and phosphorus-absorbing bacteria will also affect the removal of phosphorus. The phosphorus removal effect of the garnet matrix was stronger than that of the magnesia matrix. The decrease in the treatment effect at the higher pollutant concentrations was probably due to the matrices becoming saturated.

### 2.2. Power Generation Performance of the CW-MFC System

The system operation was divided into five stages. The two CW-MFCs based on magnesia or garnet were operated at the same time. The first stage was the establishment and trial operation of the experimental device. The cathode and anode were connected with an external 1000 Ω resistor. Simulated domestic sewage without phosphorus was introduced and the voltage was monitored. During the second stage, the two microbial fuel cell systems were operated together. The pollutant concentration was 1 mg/L total phosphorus. The pollutant concentration during the third stage was 2 mg/L total phosphorus, the pollutant concentration during the fourth stage was 3 mg/L total phosphorus, and the pollutant concentration during the fifth stage was 5 mg/L total phosphorus.

**Figure 3:**
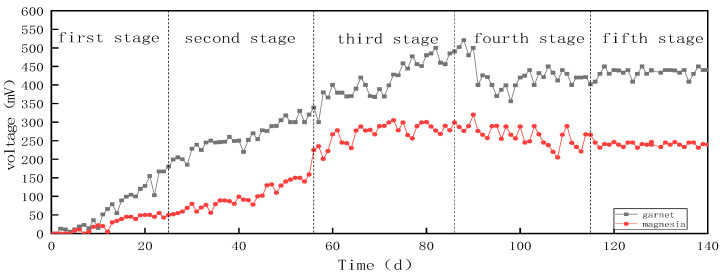
Daily voltage changes during the CW-MFC stages.

The highest output voltage and stable voltage of the pomegranate stone parts were greater than those of the magnesium devices (Figure 3). During the third stage, the total phosphorus concentration was 2 mg/L, and both devices reached their maximum output voltage. The maximum stable power generation voltage of the garnet device was 500 mV, and the maximum stable power generation voltage of the magnesia device was 290 mV.

In order to test the power generation performance of the CW-MFCs, the best power generation stage for the magnesia and garnet systems, namely the third stage, was used to conduct current density and power density tests. After the systems were stable, a 10–5000 Ω external resistor between the anode and cathode was connected and the output voltage at both ends of the external resistor was measured to obtain the system polarization curve and power density curve.

**Figure 4:**
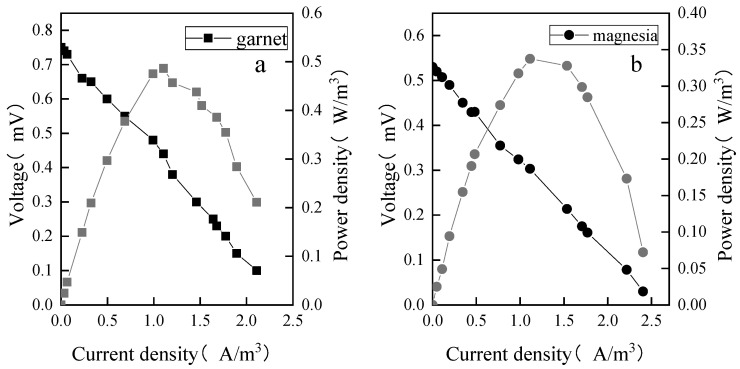
Polarization curve (black curve) and power density curve (grey curve). (**a**) Garnet matrix; (**b**) magnesia matrix.

It was found that when the open-circuit voltage of the garnet (a) matrix was 0.75 mV and the internal resistance was 245 Ω, the maximum power density was 0.48 W/m^3^, and the maximum current density was 2.1 A/m^3^ (Figure 4). The open-circuit voltage of the magnesia (b) device was 0.53 mV, the internal resistance was 450 Ω, and the greatest power density was 0.33 W/m^3^. The results show that the open-circuit voltage was determined by the internal resistance. The smaller the internal resistance was, the greater the open-circuit voltage value and the system power generation capacity were.

### 2.3. Microbial Community Structure in Wetlands

#### 2.3.1. Community Structure Characteristics of Soil Microorganisms in Wetland Sediments

**Figure 5:**
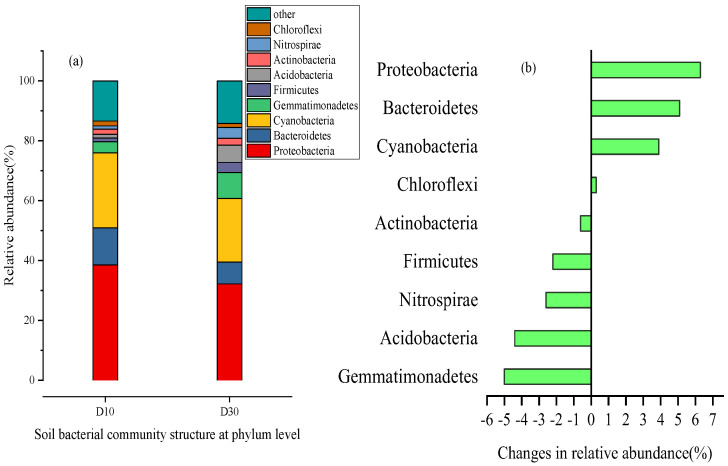
Soil microorganism changes in the wetland sediments at the phylum level. (**a**) Soil bacterial community structure at phylum level; (**b**) changes in relative abundance.

At the phylum level, there were nine dominant microbial groups with an average relative abundance of >0.1% and their relative abundance accounted for 85.8–86.6% of the total microbial community. The D10 layer mainly consisted of *Proteobacteria* (38.5%), *Cyanobacteria* (25.1%), *Bacteroidetes* (12.4%), and *Gemmatimonadetes* (3.7%), which accounted for 79% of the total microbial community. Five phyla changed significantly between the D30 layer and the D10 layer. These were *Proteobacteria* (32.2%), *Cyanobacteria* (21.2%), *Bacteroidetes* (7.3%), *Gemmatimonadetes* (8.7%), and *Acidobacteria* (5.7%). There were three phyla whose relatives decreased in the D30 layer compared to the D10 layer (Figure 5a)—*Proteobacteria*, *Bacteroidetes*, and *Cyanobacteria*—but their changes in relative abundance were positive at 6.30%, 5.10%, and 3.90%, respectively (Figure 5b). The phyla whose relative abundance increased the most between the D10 and D30 layers were *Gemmatimonadetes*, *Firmicutes*, *Acidobacteria*, *Actinobacteria,* and *Nitrospirae*, but their changes in relative abundance were −5.00%, −2.20%, −4.40%, −0.60%, and −2.60%, respectively (Figure 5b).

**Figure 6:**
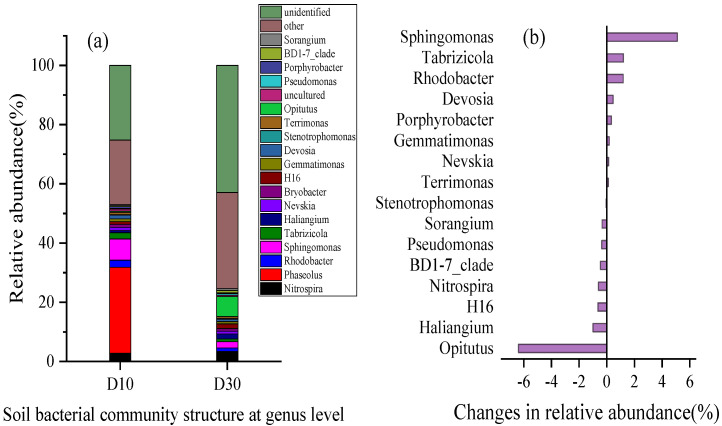
Soil microorganism changes in the wetland sediments at the genus level. (**a**) Soil bacterial community structure at genus level; (**b**) changes in relative abundance.

At the genus level, the >0.1% relative abundance groups were classified as the dominant genera. The main dominant groups in the D10 layer were *Nitrospira* (2.8%), *Rhodobacter* (2.4%), *Sphingomonas* (7.2%), *Tabrizicola* (2.1%), *Nevskia* (1.1%), and *Devosia* (1.3%) (Figure 6a). The main dominant groups in the D30 layer were *Nitrospira* (3.4%), *Rhodobacter* (1.2%), *Sphingomonas* (2.1%), *Haliangium* (1.7%), and *H16* (1.6%). The relative abundance of *Nitrospira*, *Haliangium*, *H16*, *Opitutus,* and *Pseudomonas* (Figure 6a) increased by 0.60%, 0.99%, 0.64%, 6.38%, and 0.36%, respectively. The genera that showed the most obvious decreases in relative abundance were *Rhodobacter*, *Sphingomonas*, *Tabrizicola*, *Devosia,* and *Porphyrobacter*, which decreased by 1.20%, 5.10%, 1.21%, 0.46%, and 0.34%, respectively. (Figure 6b).

#### 2.3.2. Microbial Community Structure at the Wetland Electrode

**Figure 7:**
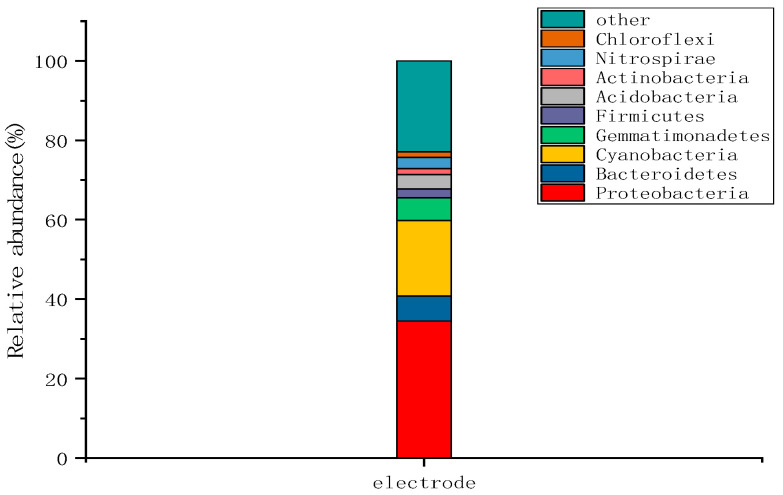
Microbial group level changes at the wetland electrode.

As shown in Figure 7, at the wetland electrode, there were nine dominant microbial groups with an average relative abundance of >1% at the phylum level, and their relative abundance accounted for 75.0–77.9% of the total microbial community. At the electrodes, *Proteobacteria* (34.5%), *Cyanobacteria* (19.0%), *Bacteroidetes* (6.3%), and *Gemmatimonadetes* (5.7%) were the main groups present, and these groups accounted for 77.1% of the total microbial community.

**Figure 8:**
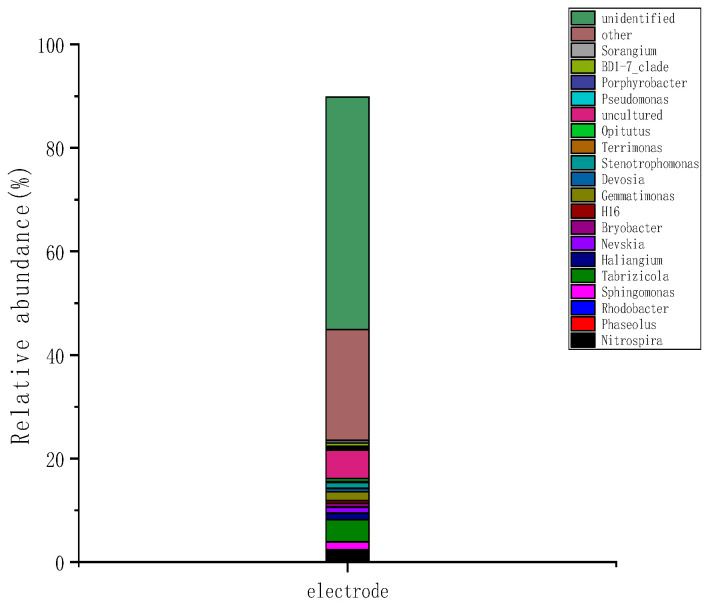
Changes in microbial genera at the wetland electrode.

As shown in Figure 8, at the wetland electrode, the groups with an average relative abundance of >0.1% at the genus level were classified as the dominant genera. The dominant genera groups were *Nitrospira* (2.4%), *Rhodobacter* (1.50%), *Sphingomonas* (4.30%), *Tabrizicola* (1.25%), *Nevskia* (0.76%), and *Devosia* (1.13%).

An analysis and comparison of the microbial community structure at the phylum level and genus level in the D10 and D30 layers of the wetland sediment soil showed that *Proteobacteria* and *Bacteroidetes* became more dominant at the phylum level, and the dominant microorganisms accounted for 85.8–86.6% of the total microbial community. At the genus level, *Haliangium* and *Opitutus* accounted for 23.29–24.23% of the microbial community. When the amounts of microorganisms in the sediment and the electrode were compared, the microbial population at the electrode was significantly lower than that in the sediment, but the migration of microorganisms became the main reason for the increase in electricity generation efficiency. The results suggest that when the CW-MFC system was operational, the significant changes in these microorganisms affected the removal of organic matter in the system and the electrical generation efficiency.

### 2.4. Analysis of the Phosphorus Removal Mechanism Used by the Two Matrices

#### 2.4.1. Scanning Electron Microscopy

**Figure 9:**
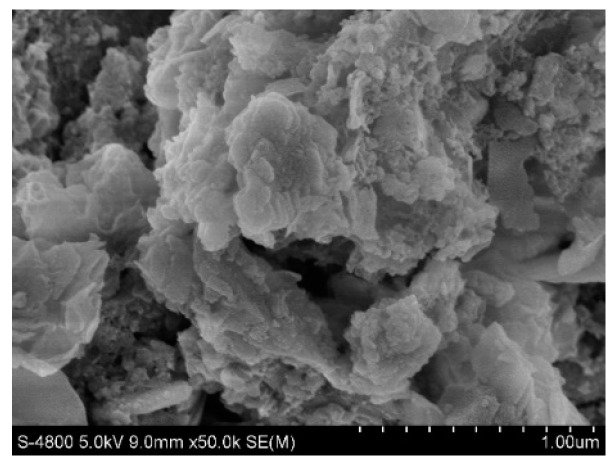
Magnesia matrix before treatment.

**Figure 10:**
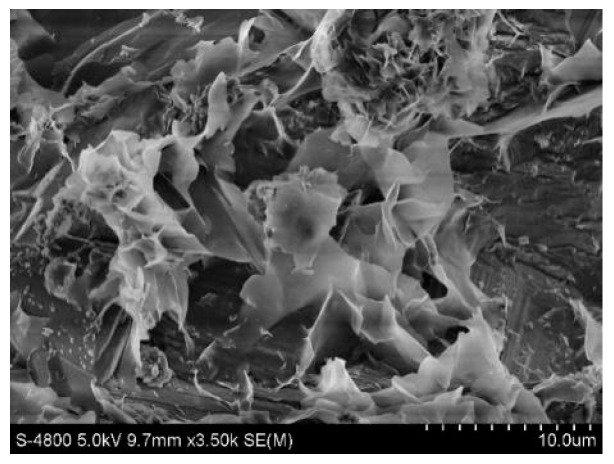
Garnet matrix before treatment.

**Figure 11:**
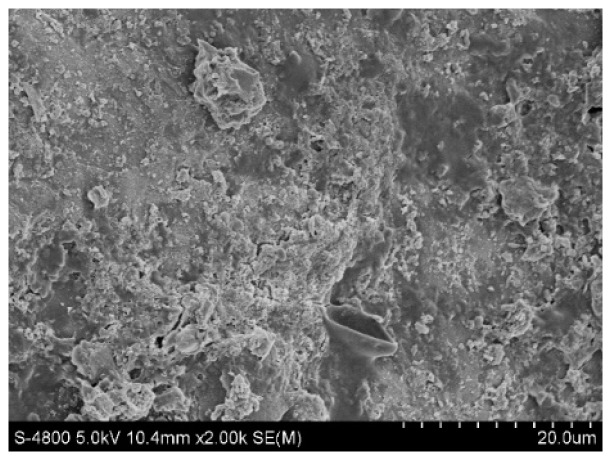
Magnesia matrix after treatment.

**Figure 12:**
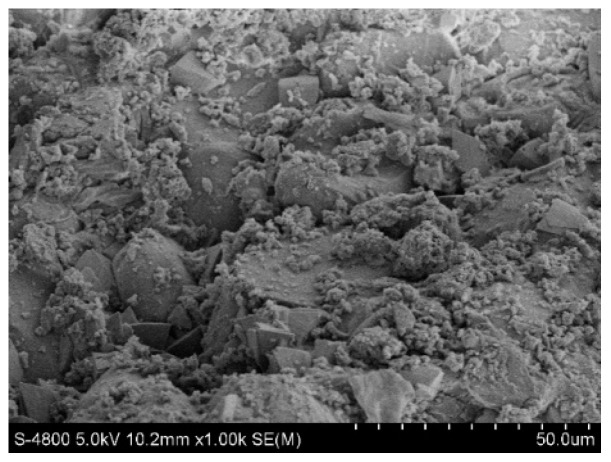
Garnet matrix after treatment.

Figure 9 and Figure 10 show the original state of the magnesia and garnet surface structures, respectively. The magnesia and garnet surfaces were relatively rough, and the magnesia surface had a typical crystal structure. A large number of small protrusions were on the surface of the magnesium oxide matrix, while a large number of lamellar structures and small pores were on the surface of the garent, making it more suitable for microbial attachment. Figure 11 and Figure 12 were electron microscopic images of the magnesia and garnet surfaces after sewage water treatment. Dense biofilms formed on the surfaces of both the magnesia and garnet matrices, and the biofilm layer on the garnet matrix surface was greater than that on the magnesia surface. This may be because the surface of the garnet matrix has more and finer pores that induce the formation of biofilm on the surface of the matrix. This biofilm plays an important role in the treatment of pollutants and enhances the effect of phosphorus degradation.

#### 2.4.2. XRD Detection and Analysis

**Figure 13:**
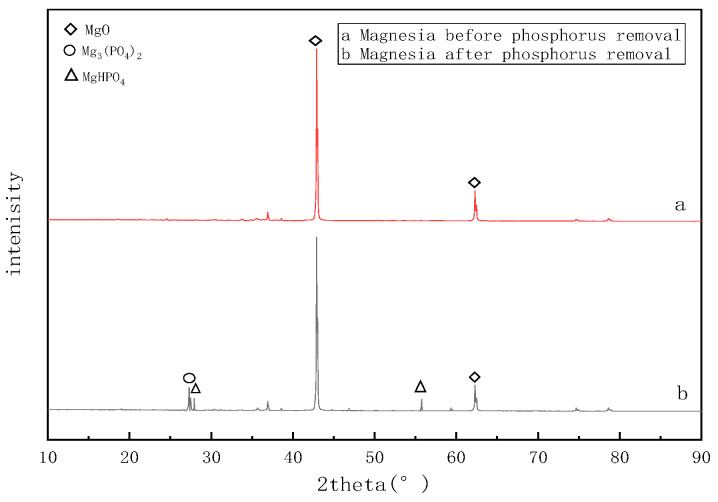
Magnesia XRD results.

**Figure 14:**
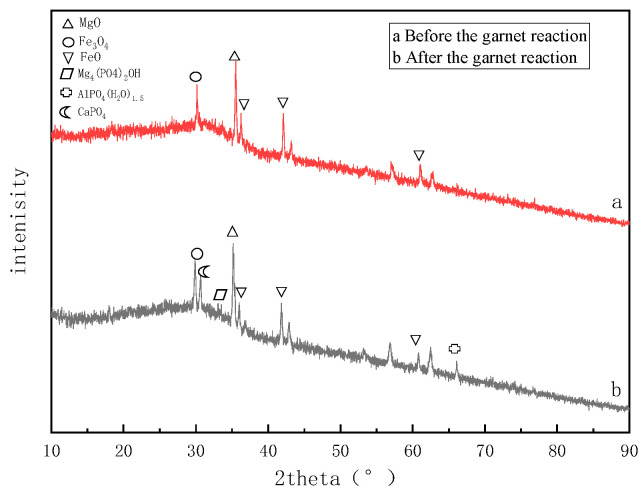
Garnet XRD results.

An XRD analysis was used to further explore the dephosphorization mechanism used by the matrices, and the object images of the matrices before and after the reaction were analyzed. The XRD images are shown in Figure 13 and Figure 14.

Figure 13 shows that the main component of magnesia was MgO. After dephosphorization, Mg_3_(PO_4_)_2_ and MgHPO_4_ appeared at 27.5° and 55.3°. Magnesium oxide dissolves in water and releases Mg^2+^ and OH^−^ and the ions diffuse outward. The precipitation forms on the surface of the substrate after the phosphate ions react with the magnesium ions from the substrate surface in the water. A large number of hydrogen phosphate ions in the alkaline water reacted with magnesium ions to form a magnesium hydrogen phosphate precipitate. Therefore, adsorption and reactions between ions may be the phosphorus removal mechanisms of magnesia.

The presence of FeO and MgO in the garnet matrix makes its composition relatively complicated. The complex adsorption process and ion exchange reactions determine the removal efficiency of phosphorus by garnet matrix similar to that of MgO. It was found that after the reaction, Mg_4_(PO_4_)_2_OH, AlPO_4_(H_2_O)_1.5_, and CaPO_4_ appeared at 33.5°, 66.7°, and 31.6°. Magnesium, aluminum, and calcium ions are all present in garnet. The phosphate and hydrogen phosphate ions in the sewage form precipitates after reacting with the ionic components that diffuse from the surface of the substrate into the water. The precipitate formed will adhere to the surface of the substrate.

## 3. Materials and Methods

### 3.1. Experimental Device

**Figure 15:**
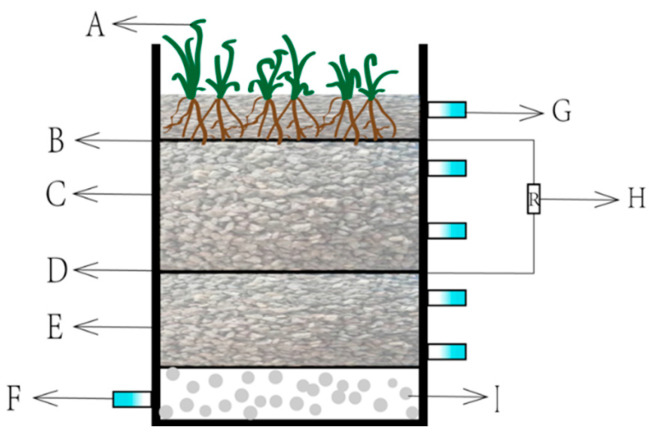
Diagram of the experimental device. A: wetland plant; B: activated carbon; C: upper packing; D: activated carbon; E: lower packing; F: water inlet; G: delivery port; H: resistance; I: gravel.

In this experiment, two types of CW-MFC devices were set up and different fillers were added. The two devices had a height of 40 cm, a length of 34 cm, and a width of 18 cm. The total volume of the apparatus was 20 L and the effective water storage volume after adding fillers was about 6 L (Figure 15).

Sampling ports were set at 9, 18, 27, and 36 cm from the bottom along the direction of the cylinder. There was a water inlet at the bottom of the device and the valve was connected to the peristaltic pump via a silicone tube. From bottom to top, the device was divided into four areas, namely the lower matrix (12 cm), anode area (8 cm), upper matrix (12 cm), and air cathode area (5 cm). Two aquatic plants were planted in the top layer. The CW-MFC system was connected to a 1000 Ω resistor, and the cathode and anode were connected via a copper wire to form a closed loop. The generated voltage was automatically measured by the data collector.

### 3.2. Selection of Experimental Materials

#### 3.2.1. Matrix Selection

The matrix plays a major role in the removal of phosphorus by the CW-MFC system. The phosphorus removal mechanism was mainly adsorption and precipitation. Studies have shown that during the phosphorus removal process in CW systems, the phosphorus removal efficiency of the substrate was much higher than that of plants and microorganisms, and the removal ratio could be as high as 70–87% [20,21]. Therefore, the selection of a suitable substrate has an important impact on the phosphorus removal efficiency of the whole system. Through kinetic experiments, three substrates were selected for control experiments, namely cordierite, garnet, and magnesia. The maximum theoretical adsorption capacity of phosphorus was 0.29 mg/g, 1.68 mg/g, and 2.33 mg/g. After comprehensive consideration and analysis of the treatment effect and economic factors, magnesia and garnet were selected as the substrates with high metal ion content and low cost. The 3–5 mm uniform magnesia and garnet were cleaned and thoroughly dried and then placed into the two devices, which made up the control group.

#### 3.2.2. Plant Selection

Plants are an important part of the microbial fuel cell system in CWs. The water plants *Eichhornia crassipes* and *Hemerocallis* were selected for their strong ability to remove phosphorus. The *Eichhornia crassipes* was bought in a flower market and the *Hemerocallis* was taken from the campus of the North China University of Science and Technology. In the two sets of devices, five uniformly sized *Eichhornia crassipes* and five *Hemerocallis* plants were placed in the middle of the air cathode and the upper substrate at the same density.

#### 3.2.3. Selection of Activated Sludge

The activated sludge was taken from the biochemical pond of the Caofeidian New Town Sewage Treatment Plant in Tangshan City, China. The treatment plant has a treatment capacity of 20,000 cubic meters per day. The equipment at the plant includes: (1) new fixed fillers in the retort; (2) new denitration deep bed filter and intermediate lifting pump room; (3) new equipment in the sludge dewatering machine room. After the sludge is retrieved, it is subjected to anaerobic treatment in the laboratory and cultured for 2 weeks. The waste was then added to the activated cathode and anode of the reactor.

#### 3.2.4. Electrode Selection

In this experiment, both the positive electrode and anode used coconut shell activated carbon as electrode material. It was granular, with developed pores, good adsorption performance, high strength, easy regeneration, and economic and durability advantages, because of its convenient use, moderate price, and wide use for drinking water, purified water, wine, beverages, industrial sewage purification, decolorization, dechlorination, and deodorization. Coconut shell activated carbon was cleaned and soaked with distilled water before use then soaked with 1 mol NaOH and HCl for 24 h. Finally, it was cleaned with distilled water more than 5 times in order to remove surface impurities. Three layers of stainless steel mesh were placed in the middle of the activated carbon to increase electrical conductivity. The upper part of the coconut shell activated carbon was a copper net exposed to the surface of the water and the air, forming the air cathode, which used polytetrafluoroethylene (PTFE) as a through film.

#### 3.2.5. Chemicals

The chemicals needed for this experiment were potassium dihydrogen phosphate, glucose, ammonium chloride, ascorbic acid, ammonium molybdate, potassium antimony tartrate, sodium hydroxide, and hydrochloric acid. A FastDNA Spin kit for soil was used to extract the soil microbial genomic DNA.

#### 3.2.6. Experimental Equipment

The main experimental equipment used included pH test paper, qualitative filter paper, measuring cylinders, a thermometer, funnels, volumetric flasks, digestion tubes, brown bottles, 50 mL colorimetric tubes with stoppers, cuvettes, test tubes, conical flasks, and beakers. The main instruments and equipment used in the experiment are shown in Table 1.

#### 3.2.7. Water Used in the Experiment

The experimental water was tap water to simulate actual domestic sewage. The theoretical water quality index is shown in Table 2 and the main compounds in the water are shown in Table 3. A trace element solution (1 mL) was added to each liter of water in the simulated sewage. The compounds and their concentrations in the trace element solutions are shown in Table 4.

The method of COD determination in the experiment was potassium dichromate rapid digestion spectrophotometry. After sampling, the water sample was filtered with medium-speed qualitative filter paper to remove impurities and suspended substances. The COD removal rate was calculated by Formula (1):

(1) $${\mathrm{COD}}_{\mathrm{remove}}=\frac{{\mathrm{COD}}_{\mathrm{in}}-{\mathrm{COD}}_{\mathrm{out}}}{{\mathrm{COD}}_{\mathrm{in}}}$$

where:

- COD concentration in influent, mg/L;
- COD concentration in effluent, mg/L.

### 3.3. Experimental Procedure

The experimental period lasted seven months, and the first phase consisted of commissioning the experiment. The plants grown in the air cathode and the upper substrate simulated the phosphorous sewage system. The trace element nutrient solution was added to the water and then pumped into the device by a peristaltic sewage pump through the bottom inlet. Then, domestication of the activated sludge and cultivation of aquatic plants began, and the COD and voltage of the effluent were monitored and recorded every day. When the COD of the effluent gradually decreased and the electricity generation voltage reached a relatively stable state, the microorganisms were successfully attached to the membrane and acclimation of activated sludge was complete. At this point, wastewater containing a low concentration of phosphorus can be introduced to start the formal experiment.

In the second stage, the CW-MFCs containing the magnesia or garnet were operated simultaneously. First, domestic sewage with a total phosphorus concentration of 1 mg/L was injected into the device, and 15 mL water samples were removed from the four outlets every 24 h, after which the total phosphorus concentrations in the water were measured after filtration through a filter membrane. The hydraulic retention time (HRT) was set to 5 days and the experiment was repeated three times. After running for one month, the total phosphorus concentration was increased to 2 mg/L, 3 mg/L, and 5 mg/L, and the above operation was repeated. Then, the different effects of the magnesia and garnet substrates on phosphorus removal were compared, and the effects of the different pollutant concentrations on the removal rate and the cathode–anode and substrate phosphorus removal efficiencies were tested. During this phase, the voltages generated by the CW-MFCs were automatically recorded by a multi-channel data collector. In the third stage, the MFC component was removed and only the CW component was retained. The second stage of the experiment was repeated to compare the pollutant removal effect of the CW and CW-MFC systems.

Soil samples were collected from the 0–10 cm and 10–30 cm layers of the CW sediments and marked as D10 and D30, respectively. A Fast DNA Spin kit for soil was used to extract soil microbial genomic DNA. A 0.5 g soil sample was weighed and the total soil microbial DNA was extracted and dissolved in 100 μL sterile TE buffer. The DNA concentration was determined by a microultraviolet spectrophotometer and DNA integrity was analyzed by 1% agarose gel electrophoresis. The soil DNA was stored in a −80 °C freezer for further analysis.

The soil DNA was amplified with bacterial universal primers 515F (5′-GTGCCAGCMGCCGCGG-3′) and 907R (5′-CCGTCAATTCMTTTRACTTT-3′). The amplification system contained 25 μL Premix TaqTM, 1.0 μL 515F primer, 1.0 μL 907R primer, and 2.0 μL 10 times diluted DNA template. Then, sterilized double-distilled water was added so that the final volume of the reaction system was 50 μL. Each PCR bacterium product was assigned a negative control of sterilized double-distilled water. The PCR amplification conditions were: 94 °C, 5 min; 94 °C, 30 s; 55 °C, 30 s; and 72 °C, 45 s over 30 cycles with a final 72 °C for 10 min. The amplification products were purified, and each sample was dissolved in 40 μL elution buffer. Then, the DNA concentration was determined again. Different samples were mixed in equal amounts and dissolved in 30 μL DNA enzyme-free H_2_O. The gel was purified with AxyPrep DNA gel recovery kit. The purified PCR products were detected by 1.8% agarose gel electrophoresis and the concentration of the purified PCR products was determined. Then, sequencing was performed on the Illumina Mi Seq sequencing platform.

### 3.4. Measurement and Calculation

Molybdenum antimony spectrophotometry was used to measure total phosphorus. Briefly, phosphate standard solutions (0, 0.5, 1, 3, 5, 10, and 15 mL) were poured into seven separate 50 mL colorimetric tubes with stoppers and distilled water was added to the 50 mL mark. Then, 1 mL 10% ascorbic acid solution was added to each colorimetric tube followed by 2 mL molybdate solution after 30 s. The tubes were mixed well and left to stand for 10 min. A spectrophotometer was used to measure the absorbance at 700 mm wavelength. Then, the pattern for the blank tube was subtracted from the results to create the standard curve for total phosphorus concentration. Subsequently, an appropriate amount of each sample was filtered via a filter membrane. After digestion, the above method was used to develop the color and measure the absorbance, and the total phosphorus concentration was obtained from the standard curve.

Voltage includes open-circuit voltage and closed-circuit voltage. In this experiment, the CW-MFC system was connected to a 1000 Ω resistor, and the cathode and anode were connected via a copper wire to form a closed loop. The data were collected every 10 min and the voltage generated was determined by equipment installed in the computer. The collector automatically recorded that this voltage was the closed-circuit voltage. Then, the cathode and anode were disconnected from the resistor and the cathode and anode wires were connected to form a closed circuit, which allowed the open-circuit voltage to be measured. The saturated calomel electrode was then connected to the open cathode and anode so that the cathode or anode potentials could be measured. The current was determined from the voltage and external resistance and calculated according to Ohm’s law.

When the external resistance changed within a certain range, the current density and the output voltage formed a curve relationship. The curve was the polarization curve and the relationship curve formed by the current and power density was the power density curve. When the system was running stably, an external resistance of 10–5000 Ω was set up between the cathode and anode so that the output voltage at both ends of the external resistance could be measured and the system polarization curve and power density curve could be obtained. These two curves were then fitted into an equation.

Quantitative PCR labeling was undertaken using universal primers 515F/907R, and general-purpose primer amplification gene cloning was used to build the gene library. The library contained the target genes in the nutrient solution. The plasmids containing the genes were subjected to plasmid purification and the plasmid concentration was determined according to the Moore constant calculation target gene copy number. Then, the plasmids were serially diluted by eight orders of magnitude to obtain the standard curves for the genes.

Three kinds of substrates were selected for the control test, namely cordierite, magnesia, and garnet. These were cleaned, dried, crushed, and sieved before use. Then, 8 g pieces of each substrate were placed in a 250 mL conical flask with a stopper, and 200 mL solutions containing 10, 20, 30, 40, 50, 60, and 70 mg/L potassium dihydrogen phosphate at neutral pH were added to the flask. The flask was placed in a constant temperature-oscillating chamber at 25 °C and 125 r/min for 24 h to equilibrate. The absorbance was determined by a spectrophotometer. The phosphorus concentration of the solution was obtained by comparing the standard curve with the spectrophotometric method. The equilibrium adsorption amount for phosphorus was calculated by the following formula.

## 4. Conclusions

The purpose of this study was to provide a reference for the selection of phosphorus removal substrates when constructing a wetland-coupled microbial fuel cell system. Overall, the system had a good phosphorus removal effect. The garnet substrate had the highest total phosphorus removal rate at 92.4%, and the magnesia substrate removal rate was 80.3%. The removal effect of the garnet substrate was better than that of magnesia, but the higher the concentration of influent pollutants, the worse the treatment effect. The biofilm layer on the surface of the garnet was greater than that on the magnesia surface. The formation of biofilm on the surface of a substrate played an important role in the treatment of pollutants and enhanced phosphorus degradation. The reason why the garnet biofilm could absorb more phosphorus was that the garnet surface had more and finer pores. Furthermore, the removal of phosphorus by the garnet matrix was mainly caused by the diffusion of the garnet components from the surface of the substrate into the water solution where they could easily react with free phosphate and hydrogen phosphate ions in the sewage, forming precipitates that attach to the surface of the substrate. The CW-MFC system showed good power generation capacity. The highest output voltage and the stable voltage of the garnet substrate were higher than those of the magnesium substrate. The devices with the two different substrates reached their maximum output voltage when the total phosphorus concentration was 460 mg/L. The maximum stable voltage of the garnet device was 500 mV and the maximum stable generating voltage of the magnesia device was 290 mV. The microorganisms in the wetland sediment soils and at the electrodes substantially changed in D10 layer, D30 layer and the electrode. *Proteobacteria* and *Cyanobacteria* were dominant at the gate level, while *Nitrospira*, *Rhodobacter*, and *Sphingomonas* were dominant at the genus level. It can be concluded that the mechanism of phosphorus removal by the matrix in the CW-MFC system is adsorption and chemical reaction between ions to generate precipitation, and the population structure of microorganisms such as *Proteobacteria* affects both electricity generation and phosphorus removal.

In this study, the garnet had a high phosphorus removal rate and could efficiently generate electricity. Future studies should investigate phosphorus removal and electricity generation by other substrates. This technology combines the advantages of constructed wetlands and microbial fuel cells while using sewage treatment and electricity output to optimize the coupling system for phosphorus removal. Therefore, when studying a CW-MFCs, the selection of electrode materials, matrix, and system structure should be taken into account to find a method that will improve the power generation capacity of the system and remove phosphorus.

## Figures and Tables

**Table 1:** List of the main instruments and equipment used in the experiment.

Equipment Name	Model	Manufacturer
Electronic analytical balance	FA2204B	Shanghai Jingke Tianmei Scientific Instrument Co., Ltd. (Shanghai, China)
Smart digester	CM-05	Beijing Shuanghui Jingcheng Electronic Products Co., Ltd. (Beijing, China)
Electric heating constant Temperature blast drying oven	DHG-9101	Jintan Medical Instrument Factory (Changzhou, China)
Magnetic stirrer	BBC-7X	Hangzhou Changsheng Group (Hangzhou, China)
UV-visible Spectrophotometer	T6	Beijing Puxi General Instrument Co., Ltd. (Beijing, China)
Peristaltic pump	YZ15	Baoding Refu Fluid Technology Co., Ltd. (Baoding, China)
Infrared spectrometer	IRAffinity-1s	Japan Shimadzu Corporation (Kyoto, Japan)
pH meter	PHS-3C	Shanghai Precision Scientific Instrument Co., Ltd. (Shanghai, China)
Ultra-pure water machine	GWA-UN	Beijing Universal. (Beijing, China)
Water bath constant temperature oscillator	THZ-82	Jiangsu Ronghua Experimental Equipment Co., Ltd. (Taizhou, China)
Multi-channel data acquisition system	PISO813	Shenzhen Changxin Automation Equipment Co., Ltd. (Shenzhen, China)

**Table 2:** Simulated sewage water quality index.

Number	Total Phosphorus Concentration mg/L	COD mg/L	Ammonia Nitrogen Concentration mg/L
1	1	230	5
2	2	460	10
3	3	690	15
4	5	1000	25

**Table 3:** COD removal rate.

Experiment Number	Factor					
Number of Groups	Output Current (A)	Current Density (mA/cm^2^)	Time (min)	pH	COD Value (mg/L)	COD Removal Rate %
1	1.199	5.995	15	2.97	37.6667	1.74
2	1.199	5.995	30	5.03	33.0000	13.91
3	1.199	5.995	45	6.95	34.6667	9.57
4	1.199	5.995	60	8.92	21.6667	19.75
5	1.599	7.995	15	5.00	36.3333	9.21
6	1.599	7.995	30	3.02	18.0000	28.95
7	1.599	7.995	45	8.99	42.0000	65.79
8	1.599	7.995	60	7.00	12.3333	54.32
9	1.906	9.530	15	7.00	23.6667	6.58
K1	0.450		0.478	0.668	4.576	
K2	1.583		1.194	1.194		
K3	1.217		1.556	1.556		
K4	1.326		1.347	1.515		
$\bar{K1}$	0.113		0.120	0.167	0.396	
$\bar{K2}$	0.385		0.299	0.299		
$\bar{K3}$	0.304		0.389	0.389		
$\bar{K4}$	0.332		0.337	0.379		
R	0.272		0.269	0.222		

**Table 4:** Trace element concentration.

Drug	Concentration (mg/L)
(NH_4_)_6_Mo_7_O_24_	0.0011
H_3_BO_3_	0.0015
FeSO_4_	0.004
CuSO_4_	0.0015
MnCl_2_	0.005
ZnSO_4_	0.022
CoCl_2_	0.0016
CaCl_2_	0.0052

## Data Availability

The experimental data used to support the findings of this study are included in the article.

## Sample Availability

Samples of the compounds are not available from the authors.
